# Impact of concurrent diabetes on periodontal health in patients with acromegaly

**DOI:** 10.1038/s41598-020-76067-5

**Published:** 2020-11-05

**Authors:** Akanksha Jain, Shipra Gupta, Anil Bhansali, Mili Gupta, Ashish Jain, Nandini Bhaskar, Rose Kanwaljeet Kaur

**Affiliations:** 1 Department of Periodontics, Dr. Harvansh Singh Judge Institute of Dental Sciences and Hospital, Panjab University, Sector-25, Chandigarh, 160014 India; 2 Unit of Periodontics, Oral Health Sciences Centre, Postgraduate Institute of Medical Education and Research, Chandigarh, India; 3 Department of Endocrinology, Postgraduate Institute of Medical Education and Research, Chandigarh, India; 4 Department of Biochemistry, Dr. Harvansh Singh Judge Institute of Dental Sciences and Hospital, Panjab University, Chandigarh, India

**Keywords:** Endocrinology, Dental diseases

## Abstract

Previous studies have suggested excess GH/IGF1 secretion in patients with acromegaly is protective for periodontal health. Diabetes is prevalent comorbidity in patients of acromegaly and is associated with worsening of periodontal disease. The present study evaluates the periodontal health and cytokines status in treatment-naive active acromegaly patients with and without diabetes. Eleven patients, each of acromegaly with and without diabetes and 20 healthy controls were enrolled. Periodontal parameters were assessed. GCF and blood samples for IL-6, TGF-β1, and PDGF were obtained. Serum GH, IGF1, HbA1c, pituitary hormones and MRI sella were performed in patients with acromegaly. There was no significant difference in periodontal status of patients with acromegaly and healthy controls. However, a significant increase in serum IL-6 (p = 0.019) and TGF-β1 (p = 0.025) levels in patients with acromegaly was observed and all patients had concurrent hypogonadism. Nevertheless, the patients with acromegaly having diabetes had modestly higher CAL and PD and serum IL-6 levels (p = 0.051), but it could not exert adverse effects on periodontal health in presence of GH/IGF1 excess. GH/IGF1 excess did not exert a protective effect on periodontal status in acromegaly, possibly due to concurrent hypogonadism and opposing cytokines; however, it could mask the ill-effects of diabetes on periodontal health.

## Introduction

Acromegaly is a rare disorder characterized by soft tissue overgrowth and proliferation as a result of increased secretion of GH/IGF1 from a somatotropinoma^1^. The annual incidence of acromegaly is 2–11 cases per million, and prevalence 28–137 cases per million^2^. The characteristic features of acromegaly include enlargement of the hands and feet, prominent supraorbital ridges, hyperhidrosis, seborrhea, and associated comorbidities like diabetes mellitus, hypertension, heart failure, and osteoarthritis^3^. Orofacial presentations include reverted and edematous fleshy lips, malocclusion, increased interdental spacing, macroglossia, prognathism, increased dental mobility, and subsequent tooth loss^4^.

Periodontitis is a chronic and progressive inflammatory disease that gradually and painlessly destroys the auxiliary tissues around the teeth and impacts the systemic health of an individual^5,6^. GH/IGF1 excess in patients with acromegaly have been shown to exhibit a protective effect on periodontal status^7–10^ through their beneficial impact on periodontal soft-tissue growth and proliferation as well on periodontal osseous tissue, namely maxilla and mandible^11^. However, patients with acromegaly are predisposed to have high prevalence of impaired glucose tolerance (IGT) and diabetes (19–56%)^1,12–14^, and dysglycaemia have a worsening impact on periodontal health of an individual^15^. Periodontal disease has been contemplated as the “sixth complication” of diabetes^16^ and both diseases (periodontal disease and diabetes) being chronic inflammatory in nature share a two-directional relationship and worsen each other^15^.

Cytokines play a pivotal role in the pathophysiology of periodontal disease. Interleukin-6 (IL-6) is a predominant proinflammatory cytokine mainly produced by polymorphonuclear leukocytes (PMNs) and is responsible for vascular modulations and migration of immuno-inflammatory cells to the periodontium^17,18^. Anti-inflammatory cytokines TGF-β and PDGF are involved in angiogenesis, cellular apoptosis, formation of proteoglycans and collagen, fibrous healing and eventually resolution of inflammation^18–20^. Besides, acromegaly comorbidities associated with it like diabetes and hypogonadism also have an impact on cytokine profile; thereby may influence periodontal outcome.

Only a few studies have explored the effect of GH/IGF1 hypersecretion on periodontal status in patients with acromegaly^7–10,21^. However, no study has assessed their effect on periodontal status in these patients with concomitant diabetes, as diabetes is a highly prevalent coexisting comorbidity and might counteract the beneficial effect of these anabolic hormones on periodontal disease in these patients. Thus, our study was planned to assess the prevalence of chronic periodontitis and levels of various anti- and pro-inflammatory cytokines in patients of acromegaly with or without diabetes.

## Methods

### Study design and subjects

Twenty-two treatment-naive patients with active acromegaly (11 with diabetes and 11 without diabetes) were enrolled in the study. All patients had clinical features of active acromegaly, presence of sweating and seborrhea elevated age and sex-matched serum IGF1, GH nadir during oral glucose tolerance test (OGTT) > 0.4 µg/l and MRI evidence of pituitary adenoma. History of hypertension and diabetes, if present, was recorded with their respective duration. The study groups comprised of Group A: acromegaly patients with diabetes; Group B: acromegaly patients with normal glucose tolerance and Group C: Twenty age and sex-matched systemically healthy adults recruited from the Department of Oral Diagnosis. Chronic smokers, pregnant women, or patients on glucocorticoid therapy/anti-inflammatory drugs/antibiotics/immuno-suppressive drugs in the previous six months or having primary hyperparathyroidism and chronic kidney disease were also excluded. Written informed consent was taken from all subjects who agreed to participate in the study after explaining the study protocol in detail. All patients of acromegaly with diabetes mellitus were treated with oral antidiabetic medication and/or insulin. All subjects underwent detailed clinical and biochemical assessment. Presence of acral enlargement, frontal bossing, sweating and seborrhea, mandibular prognathism, macroglossia, malocclusion, acanthosis nigricans, and skin tags were recorded. Visual field defects were also assessed. Full mouth intraoral periapical (IOPA) radiograms were taken of all the subjects. In addition, all enrolled subjects were asked about their diet and oral hygiene practices. Acromegaly patients were subjected to MR neuroimaging (3tesla system, Siemens, Germany) to verify the presence of pituitary adenoma. GCF and serum samples were collected from all patients and controls for the assessment of IL-6, TGF-β1, and PDGF and serum samples for growth hormone levels. Serum IGF1 levels were assessed in patients with acromegaly only (Group A and B). Postoperatively, histopathology, and immunohistochemistry for GH on tumor tissue were performed.

### Sample size calculation

Since acromegaly is a rare disease and its prevalence is 60 cases per million globally^22^, that is 0.006 per hundred. Our sample size came out to be one subject per group at a power of 95% and a confidence interval of 95%. Therefore for the application of appropriate statistical tools, it was decided to have ten subjects per group.

### Sample collection

#### GCF sample collection

GCF was collected on the next day of the periodontal assessment to avoid contamination of the sample. The sites were appropriately dried and isolated using cotton rolls to prevent salivary contamination, the supra-gingival plaque at the gingival margins was removed using a curette and a standardized volume of 4 μl of GCF was collected extra-crevicularly using color-coded 1–5 μl calibrated volumetric Hirschmann’s micro-capillary pipettes from the site with the highest loss of clinical attachment^23^. The micro-pipette was placed gently at the gingival margin without inserting it into the crevice. Any sample contaminated with blood or saliva was discarded. The sample was dispensed in Eppendorfs containing 396 μl of normal saline and stored at − 80 °C until further analysis.

#### Serum collection

Using a regular venipuncture method, 5 ml of pooled venous blood (2.5 ml at 30 min interval each) was obtained from the antecubital vein in the morning fasting state. All acromegaly patients underwent 75 g. oral glucose tolerance test (OGTT) (@0, 60 and 120 min) to assess their glycemic status and GH suppression after glucose load. The blood sample was allowed to clot at room temperature, and after 1hour, serum was removed by centrifugation. The serum was transferred to storage vials (2 aliquots) and stored at − 80 °C till required for biochemical assays^24^.

#### Biochemical analysis

GH was estimated by electrochemiluminescence immunoassay (ECLIA) (COBAS 600, Roche diagnostics, Germany). IGF1 was measured by ECLIA (Dia-Sorin, Liaison, Germany). HbA1c was estimated by HPLC using ion-exchange chromatography (Bio-Rad Laboratories, USA) with an intra-and inter-assay coefficient of variation 0.58% and 0.49%. Other hormones like serum T_4_, TSH, LH, FSH, PRL, testosterone/estradiol, and cortisol were assessed by ECLIA. All the samples were thawed only once for biochemical assessment of IL-6; PDGF, and TGF-β1 by utilizing the Sandwich ELISA technique.

#### Intraoral examination

After systemic examination and investigations, intraoral examination including Gingival Index^25^, Plaque Index (PI)^25^, Oral Hygiene Index Simplified (OHI-S)^26^, Gingival Bleeding Index (GBI)^27^ and full-mouth charting including Probing Depth (PD) and Clinical Attachment Level (CAL) were conducted for all groups. A probing depth (PD) is a dimension of the depth of a gingival sulcus or periodontal pocket. It was estimated by measuring the distance from the gingival margin to the base of the sulcus with a standardized periodontal probe (UNC-15 probe Hu-Friedy, Chicago, IL, USA). CAL is the distance from the cemento-enamel junction to the base of the periodontal pocket. A single calibrated examiner (A.J.) assessed all the clinical parameters, including full mouth probing depth and CAL. The results were reproducible when the measurements were repeated.

#### Case definition for periodontal healthy subjects and chronic periodontitis patients

Subjects presenting with BOP < 15% of sites, no CAL, PD < 3 mm, and no horizontal or vertical bone loss in the radiographic assessment at the time of dental investigations were labeled as periodontally healthy^28^. Chronic periodontitis is depicted by the formation of the periodontal pocket and/or receding gums^29^. The severity [(mild CAL 1–2 mm) (moderate CAL 3–4 mm) (severe CAL ≥ 5 mm) and magnitude [(localized presence of a well-defined pattern of the affected site or involvement ≤ 30% of teeth) (generalized expression of disease without a well-defined pattern of allocation or ≥ 30% of teeth involved)] of chronic periodontitis were categorized as per the Task Force 2015 an update to the 1999 classification of periodontal disease^28^.

### Statistical analysis

Data obtained were analyzed using Statistical Package of Social Science IBM SPSS Statistics version 23.0 (Armonk, NY: IBM Corp). Data were expressed as mean ± standard error of the mean. A p-value of less than 0.05 was considered significant. Independent ‘t’ test was used to compare periodontal status and biomarker profile in the acromegaly and control group. One-way ANOVA test was applied to see the comparison in general characteristics, periodontal status, and cytokine profile among Group A (acromegaly with normal glucose tolerance), B (acromegaly with diabetes mellitus), and C (age and sex-matched systemically healthy adults). Post hoc analyses for multiple comparisons were performed for parameters found to be significant in the ANOVA test. Further, to evaluate the effect of diabetes, an independent ‘t’ test for comparison of continuous parametric parameters and Z-test for differences in proportions between groups A and B were applied.

### Ethics approval and consent to participate

The study was performed according to the declaration of Helsinki and was approved by the Institutional Ethics Committee of Panjab University, Chandigarh, and Ethics Committee of Post Graduate Institute of Medical Education and Research, Chandigarh. Informed consent was obtained from patients to participate in the study.

### Consent for the publication

The authors confirm that they have obtained consent from the study participants to publish the data.

## Results

### Study subjects

The study subjects included 11 patients each of active acromegaly with and without diabetes and 20 healthy controls. The mean age of the patients of active acromegaly with and without diabetes was comparable with controls. More number of men had acromegaly as compared to women (13:9). Though, there was no significant disparity in the sex ratio between the two groups (Table 1).

**Table 1:** Clinical Characteristic of patients of acromegaly with and without diabetes.

Parameters	Acromegaly with diabetes (Group A)	Acromegaly without diabetes (Group B)	Controls (Group C)	P-value A vs. B	P-value A vs. C	P-value B vs. C
Number of patients	N = 11	N = 11	N = 20	–	–	–
Age (Yrs.)	41.8 ± 3.2	35.0 ± 3.7	37.2 ± 2.6	0.395	0.581	0.875
Gender (M: F)	7:4	6:5	13:7	–	–	–
Acromegaly active disease	11/11	11/11	–	1		
BMI (kg/m^2^)	28.4 ± 1.1	24.3 ± 1.5	24.0 ± 0.5	0.031*	0.007**	0.970
Mean BP [systolic/diastolic (mm/Hg)]	123.0 ± 1.0/79.0 ± 2.0	127.0 ± 4.0/80.0 ± 3.0	128.0 ± 1.0/81.0 ± 2.0	0.575/0.872	0.307/0.760	0.939/0.989
Duration of acromegaly (Yrs.)	5.4 ± 0.8	5.5 ± 0.9	–	0.623		
Acral enlargement	11/11	11/11	–	1		
Macroglossia	11/11	9/11	–	0.118		
Interdental spacing	10/11	6/11	–	0.036*		
Hypertension	3/11	4/11	–	0.646		
Hyperprolactinemia	5/11	4/11	–	0.663		
Hypogonadism	11/11	11/11	–	1		
Hypothyroidism	1/11	2/11	–	0.531		
Hypocortisolism	8/11	9/11	–	0.609		

One-way ANOVA test was applied to see the comparison among Group A, B and C. Post hoc analyses for multiple comparisons were performed for significantparameters in ANOVA test. To evaluate the effect of diabetes, independent ‘t’ test for comparison of continuous parametric parameters, and Z-test for differences in proportions between group A and B were applied.

*N* number of subjects, *Yrs.* years, *M* male, *F* female, *BMI* Body Mass Index, *BP* blood pressure.

Data was expressed as mean ± S.E.M, *p < 0.05, **p < 0.01, ***p < 0.001.

### Clinical characteristics

All patients in Group A and B had active acromegaly (Table 1).There was no age difference between the groups (p = 0.395, p = 0.581). Patients of acromegaly with diabetes had a higher BMI than acromegaly without diabetes (Group B) and controls (Group C) (p = 0.031, p = 0.007) (Table 1). However, the duration of acromegaly was similar in both the groups (A and B). All patients had active acromegaly and presented with coarse facial features and acral enlargement. Macroglossia and interdental spacing were present in 90% and 72% of acromegaly patients, respectively, while none of the healthy controls had any of these features. Moreover, there was no difference in any clinical or hormonal parameters in Group A, and B except interdental spacing (p = 0.036) (Table 1) 31.8% of the patients with acromegaly (n = 7) were hypertensive and receiving treatment for the same, while none of the controls was hypertensive. All patients with acromegaly had macrosomatotropinoma (> 10 mm) and were treatment-naïve.

### Biochemical characteristics

The mean serum fasting GH levels in patients with acromegaly with and without diabetes were 66.3 ng/ml and 64.8 ng/ml, respectively, while healthy subjects had a basal GH of 0.6 ng/ml (p = 0.008**, p = 0.010**) (Table 2). Further, the active disease status was strengthened by age and sex-matched higher IGF1 levels (778.0 and 802.9 ng/ml, 2.5-fold higher than mean) though, was comparable in acromegaly patients with and without diabetes. Patients with acromegaly had concurrent hypopituitarism due to the compressive effect of the tumor on the surrounding pituitary cells, which manifested as hypogonadism in all (100%), hypocortisolism in 17 (77%), and hypothyroidism in 3 patients (13%). Hyperprolactinemia, either as a result of stalk compression or co-secretion with GH, was present in 9 (41%) patients. Regarding other biochemical parameters, mean FPG (p = 0.000) and HbA1c (p = 0.001) levels were significantly higher in Group A as compared to those of Group B and C. The basal fasting GH (p = 0.998), nadir GH levels after OGTT (p = 0.978) and serum IGF1 levels (p = 0.185) were similar in both groups (Table 2). There was no significant difference in serum T_4,_ TSH, prolactin, testosterone/estradiol levels in both the groups, whereas serum LH and FSH levels were significantly reduced [LH (p = 0.018), FSH (p = 0.002)] in acromegaly patients with diabetes in comparison to without diabetes suggesting the effect of hyperglycemia on gonadotropes, despite similar tumor volume. Also, serum cortisol levels were significantly lower (p = 0.017) in acromegaly patients with diabetes than without diabetes (Table 2). No hormone replacement therapy was initiated before evaluations of cytokine levels in patients with acromegaly.

**Table 2:** Biochemical Characteristics of patients of acromegaly with and without diabetes.

Parameters	Acromegaly with diabetes (Group A)	Acromegaly without diabetes (Group B)	Controls (Group C)	P-value A vs. B	P-value A vs. C	P-value B vs. C
FPG (mg/dl)	140.5 ± 7.4	99.1 ± 2.0	98.1 ± 1.2	0.0001***	0.0001***	0.913
PPG (mg/dl)	195.9 ± 15.6	138.6 ± 7.4	–	0.061		
HbA1c (%)	7.8 ± 0.6	5.7 ± 0.1	–	0.001***		
GH-basal (ng/ml)	66.3 ± 16.8	64.8 ± 26.6	0.6 ± 0.2	0.998	0.008**	0.010**
IGF1 (ng/ml)	778.0 ± 101.4	802.9 ± 68.7	–	0.185		
Nadir GH-OGTT (ng/ml)	57.2 ± 14.7	47.6 ± 17.2	–	0.978		
Mean tumor volume (cm^3^)	14.5 ± 3.2	12.2 ± 4.0	–	0.375		
TSH (μU/ml)	1.06 ± 0.1	2.0 ± 1.1	–	0.090		
T_4_ (μg/dl)	6.9 ± 0.5	7.2 ± 0.5	–	0.268		
LH (mU/ml)	2.9 ± 1.0	6.7 ± 3.2	–	0.018*		
FSH (mU/ml)	2.6 ± 0.2	13.5 ± 7.0	–	0.002**		
PRL (ng/ml)	27.5 ± 6.0	21.5 ± 5.1	–	0.946		
F (nmol/l)	172.1 ± 43.4	215.5 ± 21.2	–	0.017*		
T (nmol/l)	2.8 ± 0.5	5.2 ± 0.9	–	0.69		
E_2_ (pg/ml)	4.6 ± 0.2	4.6 ± 0.2	–	0.59		

One-way ANOVA test was applied to see the comparison among Group A, B and C. Post hoc analyses for multiple comparisons were performed for significant parameters in ANOVA test. To evaluate the effect of diabetes, independent ‘t’ test for comparison of continuous parametric parameters was applied.

*FPG* fasting plasma glucose, *PPG* post-prandial glucose, *HbA1c* glycated hemoglobin, *GH* growth hormone, *IGF1* insulin like growth factor-1, *GH-OGTT* Nadir growth hormone after oral glucose tolerance test, *TSH* thyroid-stimulating hormone, *T*_*4*_ thyroxine, *LH* luteinizing hormone, *FSH* follicle stimulating hormone, *PRL* prolactin, *T* testosterone, *E2* estradiol, *F* cortisol.

Data was expressed as mean ± S.E.M, *p < 0.05, **p < 0.01, ***p < 0.001.

### Periodontal parameters

In Group A, 4 (36%) patients presented with gingivitis, 2 (18%) with mild periodontitis, 3 (27%) with moderate, and 2 (18%) with severe periodontitis as categorized based on their mean CAL levels. Following the same criteria in Group B, 5 (45%) had gingivitis, 4 (36%) had mild periodontitis, 1 (9%) patient each presented with moderate and severe periodontitis. In Group C, gingivitis was recorded for 10 (50%) subjects, mild periodontitis in 8 (40%), and moderate periodontitis in 2 (10%) subjects. There was no significant difference in periodontal parameters, including mean CAL and PD in patients with acromegaly and controls (Table 3). The mean PD was < 3 mm in all the groups; however, the mean CAL was insignificantly higher (p = 0.238 and 0.125) in patients of Group A (2.5) than Group B (1.3) and Group C (1.3) (Table 4). These mean CAL levels suggest moderate chronic periodontitis in patients of acromegaly with diabetes and mild chronic periodontitis in those without diabetes and controls (Table 4). Circulating basal growth hormone levels and CAL correlation showed a trend towards significance (r = 0.301, p = 0.053); however, no correlation with PD (r = 0.148, p = 0.349) was observed. Further analyzing the data in patients of acromegaly with and without diabetes and controls, there was no significant difference in the number of teeth present. All other parameters, including GI and PI (fair), were comparable between all the groups. However, OHI-S and GBI scores were insignificantly higher (p = 0.890 and p = 0.218) in acromegaly patients with diabetes compared to those without diabetes. There was no difference observed regarding oral hygiene and dietary habits between acromegaly and the control group.

**Table 3:** Periodontal parameters and serum and GCF cytokine levels of the study subjects.

Parameters	Acromegaly	Controls	p-value
Number of patients	N = 22	N = 20	–
Age (Yrs.)	38.4 ± 2.5	37.2 ± 2.6	0.751
GH-basal (ng/ml)	65.6 ± 15.4	0.6 ± 0.2	0.000***
IGF1 (ng/ml)	802.9 ± 84.4	–	
Number of teeth present	28.7 ± 0.9	29.8 ± 0.5	0.286
GI	1.5 ± 0.1	1.5 ± 0.1	0.516
PI	1.6 ± 0.1	1.5 ± 0.1	0.525
OHI-S	2.9 ± 0.1	3.0 ± 0.2	0.817
GBI	44.9 ± 3.8	42.9 ± 3.5	0.710
Mean PD (mm)	2.3 ± 0.1	2.1 ± 0.1	0.292
Mean CAL (mm)	1.9 ± 0.3	1.3 ± 0.3	0.194
Serum TGF-β1 (ng/ml)	80.2 ± 8.5	58.1 ± 3.4	0.025*
GCF TGF-β1 (ng/ml)	1.6 ± 0.2	1.9 ± 0.1	0.404
Serum PDGF (pg/ml)	150.5 ± 32.9	146.4 ± 28.2	0.312
GCF PDGF (ng/ml)	2.1 ± 0.2	2.9 ± 0.3	0.031*
Serum IL-6 (pg/ml)	11.5 ± 3.2	6.2 ± 0.9	0.019*
GCF IL-6 (pg/ml)	UD	UD	UD

Independent ‘t’ test was used to compare periodontal status and biomarker profile in acromegaly and control group.

*N* number of subjects, *Yrs.* years, *GI* Gingival Index, *PI* Plaque Index, *OHI-S* Oral Hygiene Index Simplified, *GBI* Gingival Bleeding Index, *PD* Probing Depth, *CAL* clinical attachment loss, *TGF-β1* transforming growth factor-β1, *PDGF* platelet-derived growth factor, *IL-6* Interleukin-6, *GCF* gingival crevicular fluid, *UD* undetected.

Data was expressed as mean ± S.E.M, *p < 0.05, **p < 0.01, ***p < 0.001.

**Table 4:** Periodontal parameters and Serum and GCF Cytokine Levels of patients of acromegaly with and without diabetes.

Parameters	Acromegaly with diabetes (Group A)	Acromegaly without diabetes (Group B)	Controls (Group C)	P-value A vs. B	P-value A vs. C	P-value B vs. C
Number of teeth present	27.8 ± 1.3	29.5 ± 1.1	29.8 ± 0.5	0.483	0.295	0.979
GI	1.5 ± 0.1	1.4 ± 0.1	1.5 ± 0.1	0.957	0.936	0.783
PI	1.6 ± 0.1	1.7 ± 0.1	1.5 ± 0.1	0.883	0.972	0.724
OHI-S	3.1 ± 0.3	2.8 ± 0.2	3.0 ± 0.2	0.890	0.996	0.898
GBI	51.3 ± 5.8	38.6 ± 4.5	42.9 ± 3.5	0.218	0.422	0.787
Mean PD (mm)	2.5 ± 0.2	2.2 ± 0.2	2.1 ± 0.1	0.375	0.250	0.996
Mean CAL (mm)	2.5 ± 0.6	1.3 ± 0.5	1.3 ± 0.3	0.238	0.125	0.991
Serum TGF-β1 (ng/ml)	67.9 ± 9.9	92.5 ± 13.2	58.1 ± 3.4	0.164	0.686	0.014*
Serum PDGF (pg/ml)	185.9 ± 70.4	224.2 ± 68.8	146.4 ± 28.2	0.892	0.855	0.547
Serum IL-6 (pg/ml)	16.6 ± 6	6.4 ± 1.5	6.2 ± 0.9	0.102	0.051	0.999
GCF TGF-β1 (ng/ml)	2.0 ± 0.4	1.2 ± 0.2	1.9 ± 0.1	0.094	0.862	0.379
GCF PDGF (ng/ml)	1.9 ± 0.2	2.4 ± 0.2	2.9 ± 0.3	0.756	0.090	0.379
GCF IL-6 (pg/ml)	UD	UD	UD	–	–	–

One-way ANOVA test was applied to see the comparison among Group A, B and C. Post hoc analyses for multiple comparisons were performed for significant parameters in ANOVA test.

*GI* Gingival Index, *PI* Plaque Index, *OHI-S* Oral Hygiene Index Simplified, *GBI* Gingival Bleeding Index, *PD* Probing Depth, *CAL* clinical attachment loss, *TGF-β1* transforming growth factor-β1, *PDGF* platelet-derived growth factor, *IL-6* Interleukin-6, *GCF* gingival crevicular fluid, *UD* undetected.

Data was expressed as mean ± S.E.M, *p < 0.05, **p < 0.01, ***p < 0.001.

### Pro- and anti-inflammatory cytokines

Pro-and anti-inflammatory cytokines including IL-6, TGF-β1, PDGF levels were assessed in serum and GCF of all subjects. A significant increase in serum IL-6 (p = 0.019) and TGF-β1 (p = 0.025) levels was observed in patients with acromegaly compared to controls (Table 3). Further, serum TGF-β1 levels were significantly higher in patients with acromegaly without diabetes (p = 0.014) as compared to controls and were non-significantly higher in patients of acromegaly with diabetes (p = 0.686) than the controls. However, serum PDGF levels were comparable in all the groups (Table 4). Proinflammatory cytokine serum IL-6 concentrations showed a higher trend in acromegaly patients with diabetes than controls (p = 0.051) (Table 4).

GCF TGF-β1 levels were higher in patients of acromegaly with diabetes than those without diabetes and controls; however, it was statistically insignificant while GCF PDGF levels were comparable in all the groups (Table 4). However, GCF IL-6 levels were undetectable in all the study groups, possibly because GCF IL-6 levels were much below the lower detection limit of the Elisa kit used. Trends varied in GCF and serum levels, which may have been due to the differences in the local and systemic expression.

## Discussion

Our study demonstrated that GH/IGF1 excess in patients with acromegaly did not exhibit a protective effect on periodontal health. However, the concurrent presence of diabetes could not exert any significant untoward effects on periodontal tissue in the presence of GH/IGF1 excess despite patients of acromegaly with diabetes had elevated levels of proinflammatory and decreased levels of antiinflammatory cytokines. High circulating levels of GH/IGF1 and concurrent presence of comorbidities like diabetes and hypogonadism in patients with acromegaly possibly abrogates the beneficial effect of GH/IGF1 on periodontal health.

Previously, various studies have demonstrated the discordant results of GH/IGF1 excess on periodontal status. A study by Lima et al. reported the complete absence of periodontal pockets in acromegaly patients compared to 50% of control subjects having periodontitis, despite 43% of their acromegaly patients having diabetes. This could be due to a different study population as 13 out of 16 acromegaly patients in their study were on octreotide therapy, and their mean GH level was 14.9 ng/ml^8^. Similarly, Serinsoz et al. reported a lower prevalence and reduced severity of periodontitis in acromegaly patients than controls irrespective of disease activity^10^. Recently a study by Ozdemir et al. also demonstrated the protective effect of GH/IGF1 in reducing periodontal destruction^9^. However, in our study, periodontal status did not differ in patients of acromegaly than healthy subjects. All 22 acromegaly patients in our study had active disease and were treatment-naïve with a cumulative GH exposure of almost 5 years. The mean GH levels were very high (65.6 ng/ml) in these patients and had two and half fold higher IGF1 levels than age and gender-matched subjects. This suggests that active disease accompanied and higher GH levels may be counter-protective on periodontal status in these patients instead of patients with relatively inactive disease and lower GH levels. Our results are in concordance with the study conducted by Bascil et al., which reported that acromegaly with the active disease showed a significantly increased frequency of chronic periodontitis compared to those with inactive or cured disease. Further, with the remission of the disease, the incidence of chronic periodontitis progressively decreased^7^.

GH has been shown to exert a ‘double-edged sword’ effect on bone health and hence, on periodontal health, as it is not only an anabolic hormone but also has a catabolic affect on bone^30,31^. These catabolic effects are more displayed on vertebrae bone as in patients with active acromegaly, vertebrae fractures occur despite an increase in bone mineral density. As vertebrae are predominantly composed of trabecular bone (cancellous) and osseous tissue of concern here, i.e. periodontal bones (maxilla and mandible) is also largely composed of trabecular bone. A recent study showed that GCF IL-1β and IL-10 levels were higher in patients with acromegaly, whereas carboxyterminal telopeptide of type 1 collagen (ICTP), a marker of bone resorption, was lower, indicating that cytokine has a predominant role in periodontal health rather than bone-associated mechanism. However, the mean GH level in this study was only 2.5 ng/ml^9^. In our study, the mean GH levels were 65 ng/ml, and these patients had relatively higher proinflammatory cytokines than antiinflammatory cytokines. Therefore, it is plausible to state that severe disease activity and higher levels of GH/IGF1 may have a detrimental influence on periodontal health in patients with acromegaly.

Associated comorbidities in acromegaly patients like hypogonadism may aggravate periodontitis; thus the expected protective effects of high serum GH/IGF1 levels on periodontitis may be diminished. Androgen/estrogen has significant anabolic and protective actions on the oral cavity. Further, maxillary and mandibular bone is predominantly cancellous (trabecular) bone, and gonadal steroids have a significant influence on bone remodeling in these bones^32,33^. These hormones enhance osteoblast proliferation and differentiation, decrease IL-6 production, and augment matrix synthesis by osteoblasts and increase osteoprotegerin levels^32,33^. Further, insufficiency of androgen/estrogen may directly affect alveolar bone irrespective of the amount of dental plaque accumulation. All our patients with acromegaly had hypogonadism, possibly due to the compressive effect of macroadenoma on surrounding gonadotropes and/or concurrent hyperprolactinemia, thereby mitigating the protective effects of GH/IGF1 on periodontal health.

Diabetes is highly prevalent comorbidity in patients with acromegaly^12–14^. In our study, patients of acromegaly who had diabetes were older and had a higher BMI than acromegaly without diabetes regardless of GH/IGF1 levels and tumor volume. Both advancing age and higher BMI attribute to increased insulin resistance and subsequent development of diabetes. In addition, GH-mediated glycogenolysis and gluconeogenesis lead to increased hepatic glucose output and further worsens hyperglycemia. High blood glucose level has been shown to exert detrimental effects on periodontal health. This has been attributed to increased accumulation of advanced glycated end products, altered polymorphonuclear neutrophil function, concurrent microangiopathy, and enhanced release of various proinflammatory cytokines, which subsequently lead to increased periodontal tissue breakdown^6^. In our study, patients of acromegaly with diabetes had higher CAL and PD, though non-significant, from acromegaly without diabetes and had significantly higher levels of IL-6. Therefore, this is conceivable to conclude that the worsening effect of hyperglycemia on periodontal health in these patients was blurred by the concurrent presence of GH/IGF1 excess.

Cytokines are signaling proteins that have a role in the expression of host immune response to complex pathogenic bacteria. Results of our study show significantly higher serum TGF-β1 levels in acromegaly patients than controls. GCF TGF-β1 levels were higher in patients of acromegaly with diabetes than those without diabetes and controls; however, it was statistically insignificant. Trends varied in GCF and serum levels of cytokine, which may have been due to the differences in the local and systemic expression. TGF-β1 induces pre-osteoblastic cell multiplication, collagen formation, bone matrix synthesis, and enhances the activity of alkaline phosphatase. Our results are in consonance with Bolanowski et al., who demonstrated higher concentrations of TGF-β1 in male patients with active acromegaly than the cured ones^34^. Platelet-derived growth factor (PDGF) favors migration and proliferation of fibroblasts, osteoblasts, and alveolar bone cells^20^. In our study, patients of acromegaly with mild to moderate periodontitis had elevated PDGF levels, which is an adaptive response to ongoing damage to periodontal tissue, while GCF PDGF levels were comparable in all the groups. To the best of our knowledge, no other study to date has evaluated serum and GCF PDGF levels in patients with acromegaly.

In our study, the serum IL-6 concentrations were significantly higher in acromegaly patients than controls. These findings are similar to a study by Andreassen et al., which concluded a structural similarity between IL-6 receptors and GH receptors but exert a contrary effect on periodontal health^35^. Further, in their study, they demonstrated that GH treatment led to an increase in blood glucose levels, which in turn resulted in increased IL-6 levels^35^. This explains the significantly raised serum IL-6 levels in acromegaly patients with diabetes as compared to without diabetes in our study. Moreover, concurrent testosterone deficiency in acromegaly patients could be another mechanism leading to raised serum IL-6 levels as testosterone inhibits prostaglandin secretion and IL-6 synthesis (Güncü et al.; Parker et al.)^33,36^. Our results are, however in contrast to those reported by Ueland et al., who reported no change in serum IL-6 concentrations of acromegaly subjects than controls^37^. This difference could be due to the varying subject population. In their study, only 17 out of 47 acromegaly patients had hypogonadism, while all acromegaly subjects in our study had hypogonadism, and moreover, their patients had lower GH and IGF1 levels as compared to our patients.

The strengths of our study include assessment of periodontal health in a rare disease like acromegaly and evaluation of cytokines levels in these subjects. However, our study has some limitations, including cross-sectional design, a smaller number of patients, and failure to assess the impact of hyperglycemia and hypogonadism in these patients. Moreover, estimation of bone turnover markers would help broaden the existing knowledge regarding the underlying pathogenesis of the periodontal disease process in acromegaly patients.

## Conclusion

In conclusion, GH/IGF1 excess did not exert a protective effect on periodontal status in patients with active acromegaly, possibly due to concurrent hypogonadism and opposing effects of proinflammatory and antiinflammatory cytokines. However, GH/IGF1 excess could mask the ill effects of diabetes on periodontal health.

## Data Availability

The datasets supporting the conclusions of this work are included in the article. On reasonable requests, the content can be available from the corresponding author.
